# Neuroprotective effect of cedrol in a male rat model of Parkinson's disease

**DOI:** 10.14814/phy2.70309

**Published:** 2025-04-07

**Authors:** Fatemeh Forouzanfar, Mahmoud Hosseini, Amir Mahmoud Ahmadzadeh, Ali Mohammad Pourbagher‐Shahri

**Affiliations:** ^1^ Neuroscience Research Center Mashhad University of Medical Sciences Mashhad Iran; ^2^ Department of Neuroscience, Faculty of Medicine Mashhad University of Medical Sciences Mashhad Iran; ^3^ Division of Neurocognitive Sciences, Psychiatry and Behavioral Sciences Research Center Mashhad University of Medical Sciences Mashhad Iran; ^4^ Department of Radiology, School of Medicine Mashhad University of Medical Sciences Mashhad Iran

**Keywords:** cedrol, herbal medicine, neurodegenerative disorder, Parkinson disease, sesquiterpene

## Abstract

Cedrol is a natural sesquiterpene subgroup of terpenes with antioxidant and anti‐inflammatory properties. This study evaluated the effect of cedrol on the 6‐hydroxydopamine (6‐OHDA) rat model of Parkinson's disease (PD). Unilateral injection of 6‐OHDA was performed to induce the PD model. Cedrol at 10 mg/kg and 20 mg/kg was administrated. The rotarod test, apomorphine‐induced rotational test, and open field tests assessed motor function, while the passive avoidance test was used to evaluate cognitive function. Furthermore, striatal levels of malondialdehyde (MDA) and total thiol, and SOD (superoxide dismutase) activity were measured. The induction of lesion led to a significantly higher number of rotations in the apomorphine‐induced rotational test, lower maintenance in the rotarod test, as well as a shorter delay for entering into and a longer duration of time spent in the dark chamber in the passive avoidance test, versus the control group. It also enhanced the striatal levels of MDA and diminished the striatal SOD activity and level of total thiol. Administration of cedrol significantly improved behavioral tests and biochemical assays. Cedrol could benefit cognitive and motor functions in a rat model of PD. Its antioxidant properties might mediate these effects.

## 1 INTRODUCTION

Parkinson's disease (PD) is a complex, progressive, and chronic neurodegenerative disorder. Prevalence and disability caused by PD have rapidly grown in recent years (Ou et al., 2021). The incidence of PD increases in older ages (Ou et al., 2021; Simon et al., 2020), with most cases happening to those over 60. Although prevalence estimates vary by country, it is generally estimated to impact 1%–2% of people over 65 (Tenchov et al., 2025).

The upward trend in population age leverages the issue of rapid changes in PD prevalence and underscores the need for investigating efficient therapeutic methods (Simon et al., 2020). The worldwide prevalence of Parkinson's disease has doubled over the past 25 years and now reaches more than 8.5 million people (Crooks et al., 2025).

Dopamine depletion in the nigrostriatal pathway appears to be the cause of PD. In PD, intracytoplasmic inclusions known as Lewy bodies of dopamine neurons are frequently found. One of the main causes of the illness is the degradation of dopaminergic neurons in the substantia nigra compacta, which is followed by decreased dopamine release in the striatum (Salaramoli et al., 2023, 2024). The death of dopaminergic cells in the substantia nigra compacta has been attributed to several theories, including mitochondrial complex malfunction, inflammatory immunological responses, and increased reactive oxygen species (ROS) generation (Rabiei et al., 2019). ROS generation, which is also a hallmark of aging, can endanger the viability of dopaminergic neurons and interfere with their normal function (Aborode et al., 2022). PD is typically characterized by motor symptoms, the most prominent of which are resting tremor, rigidity, and bradykinesia. This is because the primary site of involvement in PD is substantia nigra pars compacta, where the largest number of dopaminergic neurons reside (Bigham et al., 2021). The dopamine secreted by these neurons is essential for controlling movements. Non‐motor symptoms, such as cognitive decline, can also present during the disease. Noteworthy, the emergence of dementia has been reported to be significantly associated with mortality rates in PD patients (Gonzalez‐Latapi et al., 2021). The focus of routinely applied treatments is on alleviating patients' symptoms rather than preventing progressive neuronal degeneration. Levodopa, alone or in combination with dopamine receptor agonists and catechol‐O‐methyltransferase inhibitors or monoamine oxidase‐B, is frequently prescribed as the mainstay of treatment. Patients experience good responses to this therapeutic regimen at early stages; however, as the disease progresses, its efficacy is reduced, and unwanted effects, such as behavior changes, dyskinesia, and motor fluctuation (Silva et al., 2022). This highlights the need for investigation of new efficient therapeutic approaches. The majority of studies against PD focus on possible targets that may offer neuroprotection, reduce inflammation in the brain or neutralize mediators linked to the disease's etiology. The first involves compounds with specific effects on neuronal tissue that have antioxidant properties; the second involves studies on components that have anti‐inflammatory properties; and the third group involves components that have effects on the dopaminergic system, either as agonists or as compounds that avoid or reduce dopamine metabolism. Furthermore, substances that impede the function of muscarinic or ionotropic glutamate receptors in the central nervous system include a fourth category (Ríos et al., 2016).

Various plants have been reported to possess therapeutic properties attributed to their bioactive components (Salmerón‐Manzano et al., 2020). Among these components, terpenes constitute the most diverse group, which are categorized based on the number of isoprene units (Cox‐Georgian et al., 2019; Forouzanfar et al., 2022). C‐15 terpenoids are called sesquiterpenes. These are significant components of essential oils, which are used in soap and fragrance formulations in addition to a variety of medical purposes. Additionally, they can be found in aroma combinations as flavoring chemicals (Merfort, 2002). Cedrol is a natural sesquiterpene, a subgroup of terpenes, that has antioxidant and anti‐inflammatory properties (Asgharzade et al., 2025; Forouzanfar et al., 2022). Furthermore, it has been revealed that cedrol could modulate some neurotransmitters in the brain, including dopamine (Zhang & Yao, 2019). According to these, we hypothesized that cedrol might be advantageous for PD treatment.

This study aimed to evaluate the effect of cedrol on the 6‐hydroxydopamine (6‐OHDA) rat model of PD.

## 2 MATERIALS AND METHODS

### 2.1 Chemicals

Ketamine and xylazine were bought from Alfasan Pharmaceutical Co. (Woerden, Netherlands). 2‐thiobarbituric acid (TBA), 3‐[4,5‐dimethylthiazol‐2‐yl]‐2,5 diphenyl tetrazolium bromide: MTT, 5–5′ dithio‐bis‐2‐nitrobenzoic acid: DTNB, dimethyl sulfoxide (DMSO), ethylenediaminetetraacetic acid (EDTA), pyrogallol, hydrochloric acid (HCl), trichloroacetic acid (TCA), potassium chloride, Apomorphine (A‐4393) 98.5%, 6‐hydroxydopamine (162957) 95% (HPLC), and Cedrol (22135) 99.0 GC were bought from Sigma‐Aldrich (Sigma Aldrich St Louis MO). Rat food was prepared by Behparvar Company (Iran).

### 2.2 Animals

Rats (Wistar, male, 200–250 g) were supplied by the experimental animal center of Mashhad University of Medical Sciences (MUMS), Mashhad Iran. To perform the experiments of this study, the rats were placed in standard cages and kept in controlled laboratory conditions. Rats were exposed to light cycles for 12 h and dark cycles for 12 h. The temperature and relative humidity were adjusted to 22–24°C and 60 ± 5%, respectively. During the study period, free access to food and water was provided to the rats under study.

The approval of the Institutional Animal Ethics Committee was obtained for the study, and all studies were conducted in compliance with the guidelines for the care and use of laboratory animals (No: 4010185, IR.MUMS.AEC.1401.039).

### 2.3 Induction of PD

To induce PD, the rats were anesthetized using xylazine (10 mg/kg) and ketamine (100 mg/kg) with intraperitoneal injection. They were then fixed in a stereotactic device, and the skull was exposed. In the next step, 6‐OHDA (16 μg/4 μL 0.2% ascorbate saline) was injected in the left MFB by a Hamilton syringe (2 μL/min) at the following coordinates: anteroposterior: −3.6 mm; mediolateral: +1.8 mm; dorsoventral: −8.2 mm (Bigham et al., 2021). The syringe was gently removed from the injection site, and the incision was sutured and disinfected. The rats were ultimately returned to their cages after recovery from surgery. All surgical procedures were performed under aseptic conditions.

### 2.4 Grouping

**FIGURE 1:**
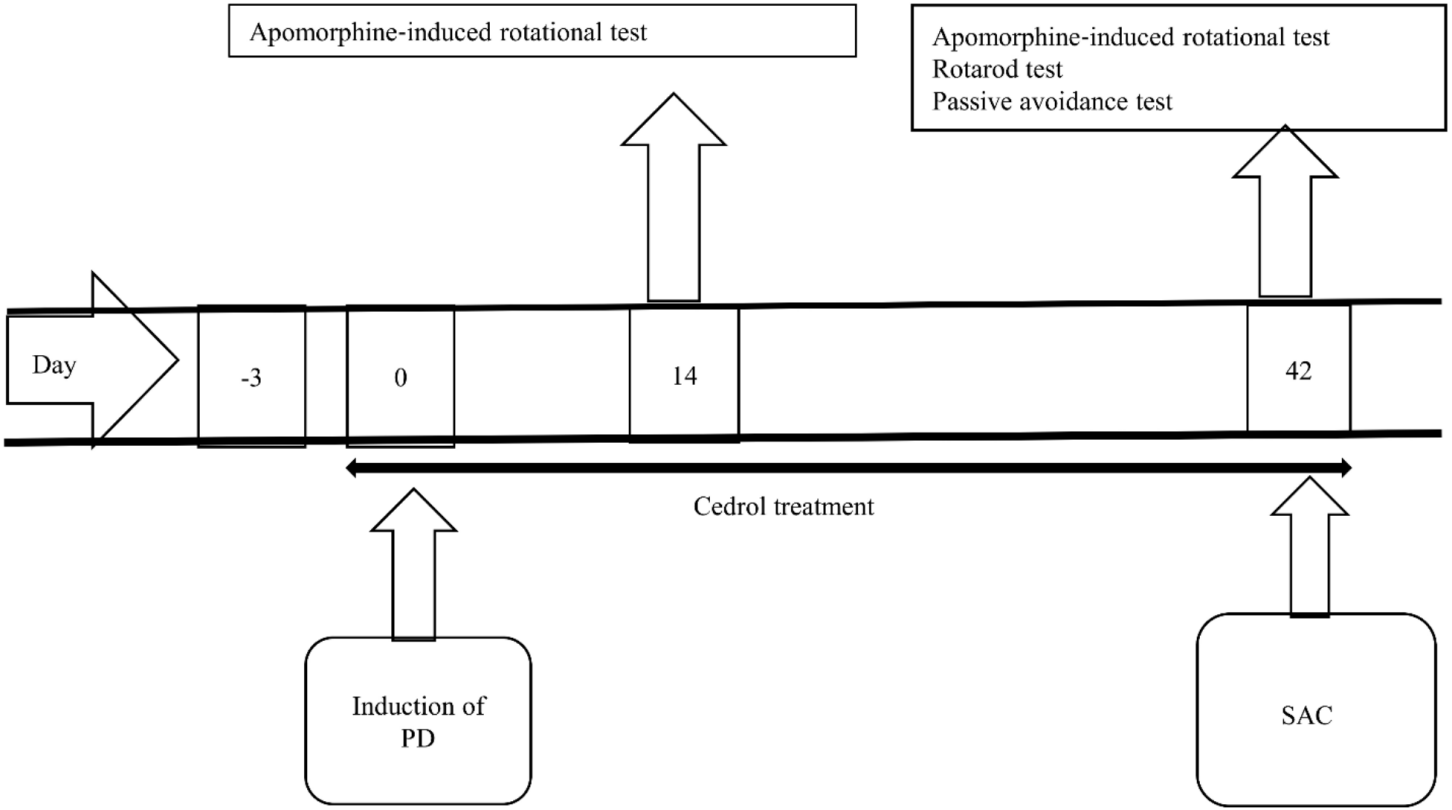
Diagrammatic sketch for the behavioral and biochemical experiments. PD, Parkinson's disease; SAC, sacrificed for biochemical experiments. Day 0 refers to the day of surgery.

The rats were divided into four groups of eight each. The low‐dose cedrol group took an oral administration of 10 mg/kg of cedrol, while the high‐dose cedrol group received an oral administration of 20 mg/kg of cedrol. Rats in the control group and Parkinson's group received oral administration of normal saline. All the treatments were applied from 3 days before surgery until 6 weeks' post‐surgery (daily). Figure 1 shows a diagrammatic sketch for the experiments.

All vehicles and drugs were accurately administered through oral gavage. Cedrol was dissolved in saline with a concentration of 1% DMSO. All assessments were conducted in a blinded manner, and the researcher was unaware of which treatment each rat had been given.

### 2.5 Apomorphine‐induced rotational test

This test represents the extent of damage to the nigrostriatal pathway. For performing this test, the animals were acclimated in plexiglass boxes for 10 min. Then, 2 mg/kg of apomorphine was injected intraperitoneally, and after 1 min, the number of rotations was documented at 10‐min intervals for 60 min. The net number of rotations was calculated by subtracting ipsilateral rotations from contralateral rotations. Rats with equal to or more than 7 contralateral rotations/min were considered appropriately lesioned. The test was performed 2 and 6 weeks post‐surgery (Bigham et al., 2021).

### 2.6 Rotarod test

To assess balance and motor coordination, a rotarod test was performed. Before the experiment, the rats were placed on a rod rotating at the speed of 10–40 rpm for 3 days to be acclimated to the environment. On the day of the experiment, the rats were placed on the device again and the time that passed until the animal fell off the rotating rod was documented for each rat. The maximum time was set at 300 s (Shirzad et al., 2023).

### 2.7 Passive avoidance test

For assessing cognitive function, we performed a passive avoidance test 6 weeks post‐PD induction. A Shuttle box with two bright and dark rooms and a connecting door was utilized for this purpose. Before the induction of PD, the rats were placed in the bright chamber. Upon entrance to the dark space, the door was shut and a shock (2 mA, 2 s) was delivered to the animal's foot. The rats were placed in the bright room 1, 2, and 3 days after they received the first shock. The duration before entering the dark chamber (delay time), as well as the duration spent in the dark space, was recorded (Forqani et al., 2024).

### 2.8 Open field test

This test was run to evaluate anxiety‐related behaviors and locomotor activity. There are 16 block series that make up the open field aperture. The inner zone (four blocks) was regarded as central, and the outside zone (12 blocks) as peripheral. In the fourth week following injection, each animal was placed individually in the center section of the box. Every rat's movements were captured for 10 min with a digital camera, and the crossing, distance, and time in the central and peripheral zones were recorded (Bigham et al., 2021).

### 2.9 Measurement of oxidative stress markers

Following the completion of final behavioral tests, rats were scarified through CO_2_ inhalation, then decapitated, and their brains removed; the striatum was separated on dry ice. The striatal was kept at −80°C for further experiments. The striatal was homogenized with PBS (pH 7.4).

### 2.10 Estimation of MDA levels

Briefly, the homogenate sample was heated with the TCA‐TBA‐HCl solution in a boiling water bath for 20 min to develop a colored adduct. Then it was cooled and centrifuged for 10 min at 3000 rpm. The supernatant was poured into the plate, and the absorbance was read at a wavelength of 535 nm. Tissue levels of MDA were calculated as nmol/g tissue (Asgharzade et al., 2025).

### 2.11 Estimation of total thiol levels

Tissue homogenate was mixed with a solution containing TCA + EDTA, and then the absorbance of this mixture was measured at 412 nm against Tris‐EDTA buffer alone (A1). After adding 20 μL of DTNB reagent (10 mM) to tubes (A2), the absorbance was measured at 412 nm to determine the second absorbance. The absorbance of the DTNB reagent as a blank solution was read at 412 nm (B). The following formula (mmol/mg tissue) was used to determine the tissue levels of total thiol: (A2 – A1 –B) × 1.07/0.05 × 13.6 (Asgharzade et al., 2025).

### 2.12 Estimation of SOD activity

SOD activity was estimated using the method that Madesh and Balasubramanian outlined (Madesh & Balasubramanian, 1998). The autoxidation of pyrogallol produces superoxide, and SOD prevents the superoxide‐dependent reduction of the tetrazolium dye to its formazan, leading to a colorimetric assay. At 570 nm, the changes in colorimetric readings were recorded. The quantity of enzyme that results in 50% inhibition of the tetrazolium dye (MTT) reduction rate is considered one unit of SOD activity (Mokhtari‐Zaer et al., 2020).

### 2.13 Statistical analysis

Analysis of data was calculated by SPSS (version 26) and the graphs were made by Graph Pad Prism (version 8.0) software. Kolmogorov–Smirnov test was used to evaluate normality. For comparing data between the groups, one‐way ANOVA and Tukey's post hoc tests were used. As nonparametric test Kruskal–Wallis followed by Dunn's pairwise comparison test was used. All The data were represented as mean ± SD. The data of apomorphine‐induced rotation, passive avoidance, and open field tests were presented as median and interquartile range. *p* value <0.05 was considered statistically significant.

## 3 RESULTS

### 3.1 Apomorphine‐induced rotational test

The results showed that there was a significant difference among the groups on the number of rotations on the 2 weeks (H_(2)_ = 19.29, *p* = 0.000) and 6 weeks (H_(2)_ = 19.73, *p* = 0.000).

**FIGURE 2:**
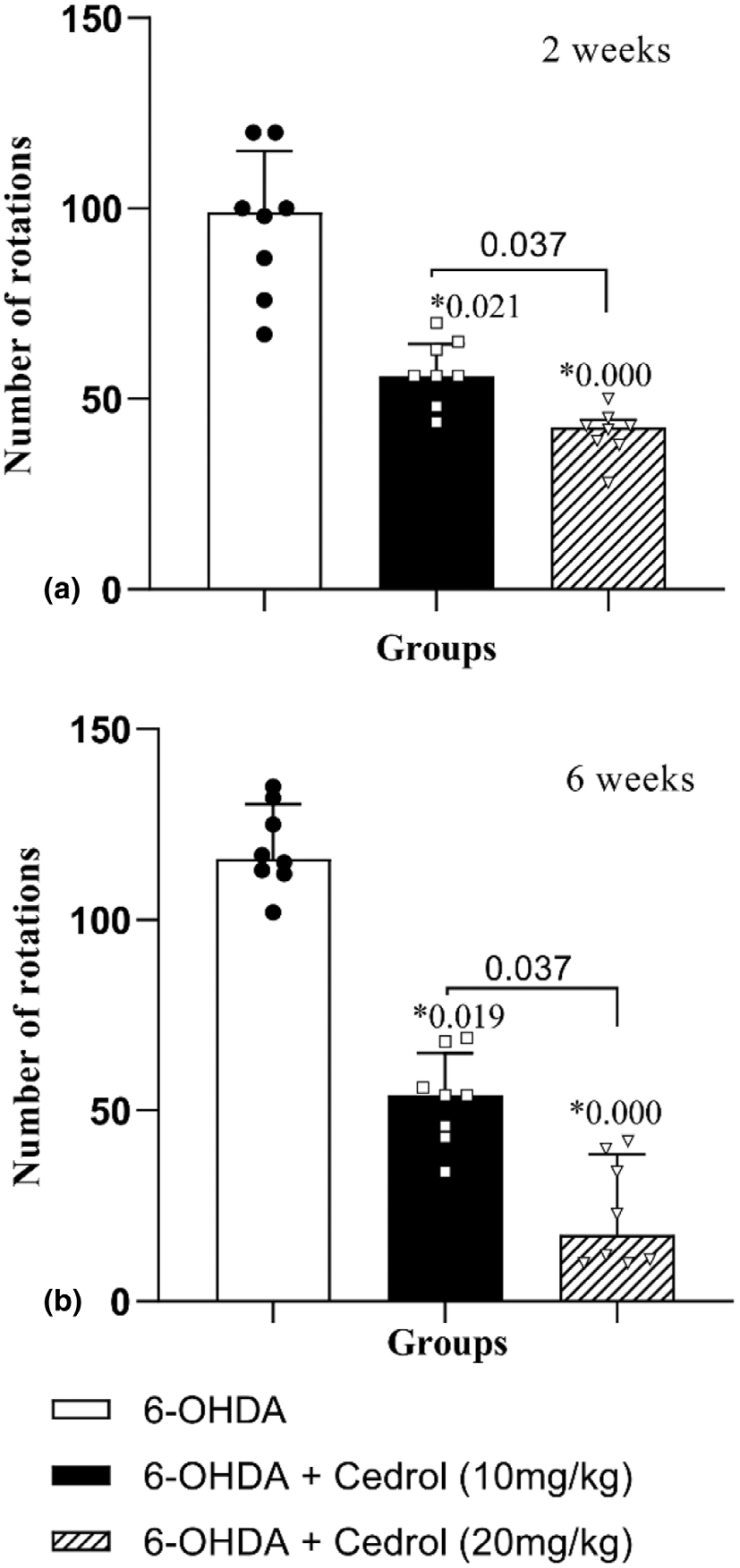
The effects of cedrol on the number of rotations in the apomorphine‐induced rotational test. *Compared to the Parkinson group. The data were presented as the median and interquartile range (*n* = 8 in each group).

Treatment with either dose of cedrol diminished the mean number of rotations at both 2‐ and 6‐week assessments, versus the PD group (45.57% and 57.29% of PD group for low and high doses of cedrol, respectively, at 2 weeks and 55.41% and 80.86% of PD group for low and high doses of cedrol, respectively, at 6 weeks) (*p* = 0.021 and *p* = 0.000 for low and high doses of cedrol, respectively, at 2 weeks and *p* = 0.019 and *p* = 0.000 for low and high doses of cedrol, respectively, at 6 weeks). The effects of cedrol was dose dependent at both 2 (*p* = 0.037) and 6 weeks (*p* = 0.037) (Figure 2).

### 3.2 Rotarod test

**FIGURE 3:**
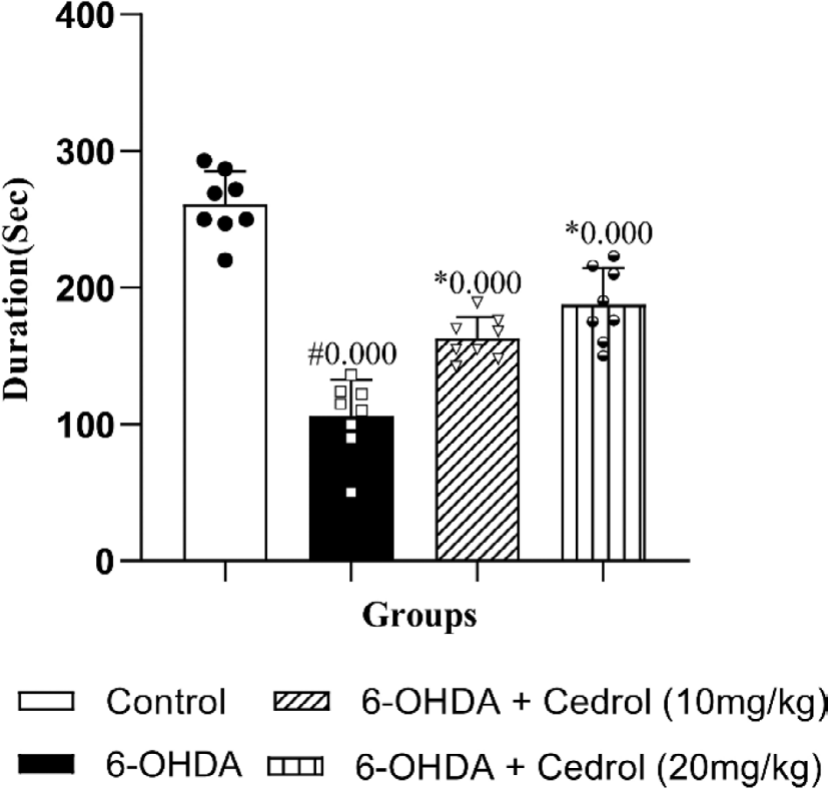
The effects of cedrol on the duration spent on the rotating rod in the rotarod test. #Compared to the control group, *Compared to the Parkinson's group. The data were presented as mean ± SD (*n* = 8 in each group).

The results showed that there was a significant difference between the groups on the time the rats spent on the rotating rod over the 6 weeks (*F*_(3,28)_ = 58.377, *p* = 0.000). Administration of 6‐OHDA significantly attenuated the time the rats spent on the rotating rod in the PD group versus the control‐operated rats (59.43% reduction compared to the control group) (*p* = 0.000). This duration significantly increased in both treatment groups (*p* = 0.000 for both) versus the PD group (53.83% and 77.10% compared to the PD group) (Figure 3).

### 3.3 Passive avoidance test

**FIGURE 4:**
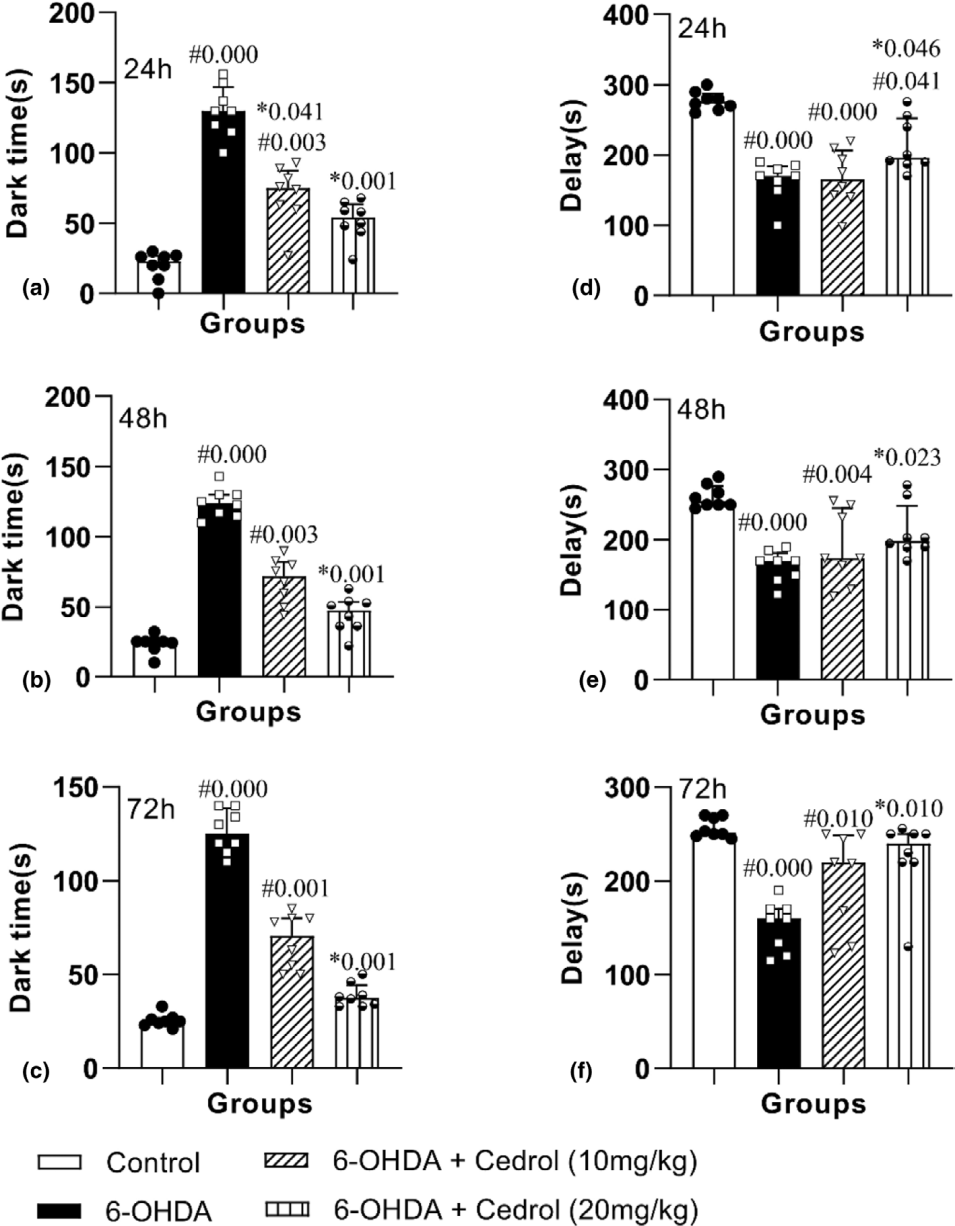
The effects of cedrol on the duration spent in the dark chamber (a) 24 h, (b) 48 h, and (c) 72 h, the duration of delay before entering the dark chamber (d) 24 h, (e) 48 h, and (f) 72 h in the passive avoidance test. #Compared to the control group, *Compared to the Parkinson's group. The data were presented as median and interquartile range (*n* = 8 in each group).

The results showed that there was a significant difference between the groups on the time spent in the dark chamber in the 24 h (H_(3)_ = 26.17, *p* = 0.000), 48 h (H_(3)_ = 26.57, *p* = 0.000), and 72 h (H_(3)_ = 28.79, *p* = 0.000). Regarding the time spent in the dark chamber, induction of the lesion significantly prolonged the dark time durations in the PD group at 24 (552.99%), 48 (431.10%), and 72 h (394.58%) versus the control‐operated rats (*p* = 0.000 for all). Administration of the highest dose of cedrol significantly shortened the durations in 24 (59.92%), 48 (63.94%), and 72 h (69.92%) (*p* = 0.001 for all) versus the PD group (Figure 4a–c). The results showed that there was a significant difference among the groups on time delay for entering the dark room on the 24 h (H_(3)_ = 20.68, *p* = 0.000), 48 h (H_(3)_ = 16.89, *p* = 0.001), and 72 h (H_(3)_ = 17.51, *p* = 0.001). Time delay for entering the dark room was significantly lower in the PD group at 3 time points (41.00, 37.79, and 4062) (*p* = 0.000 for all) versus the control group. Only treatment with the high dose of cedrol led to a significant increment of the delay time at 24 h, 48 h, and 72 h (*p* = 0.046, 0.023, and 0.010 for 24 (30.80%) and 48 (30.05%) and 72 (48.15%) h, respectively) versus the PD group (Figure 4d–f).

### 3.4 Open field test

**FIGURE 5:**
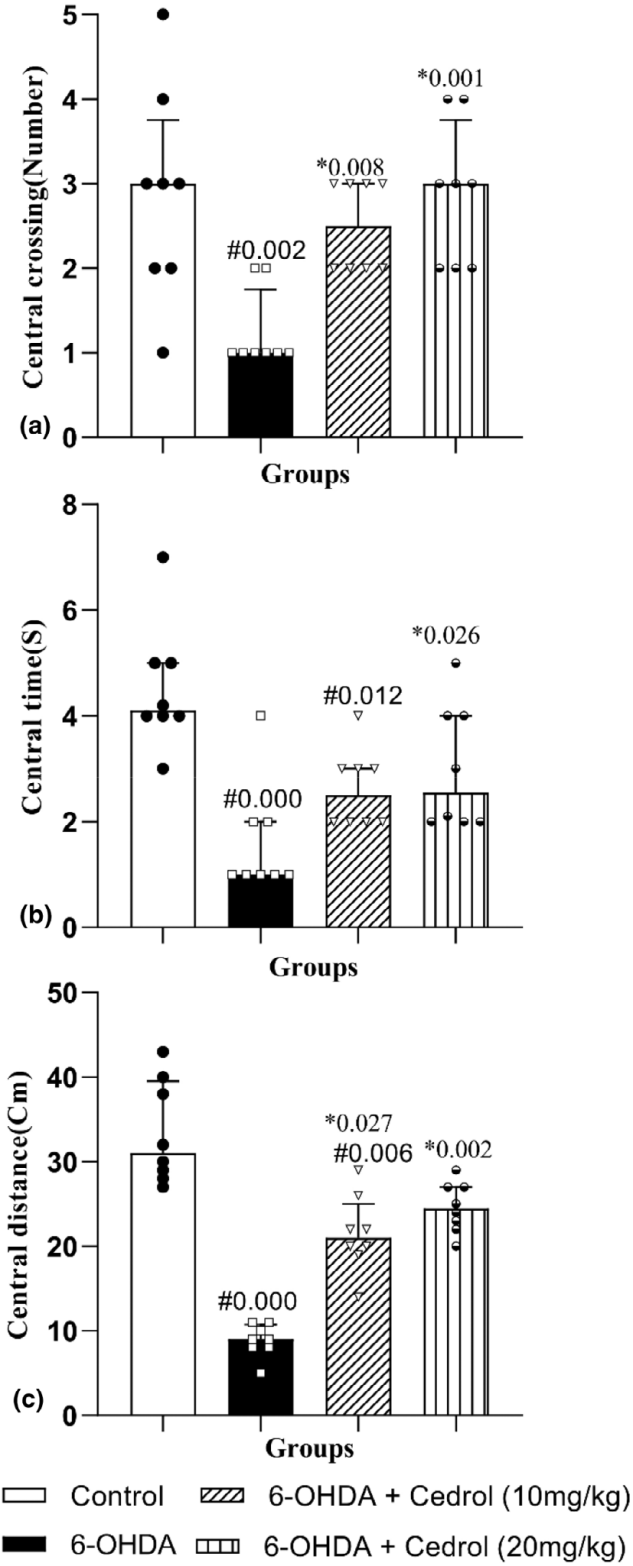
The effects of cedrol on the central crossing number (a), the time spent in the central zone (b), and the central traveled distance (c), of the open field test. #Compared to the control group. *Compared to the Parkinson's group. The data were presented as median and interquartile range (*n* = 8 in each group).

The results showed that there was a significant difference between the groups on central crossing (H_(3)_ = 14.31, *p* = 0.003), central time (H_(3)_ = 17.30, *p* = 0.001), and central distance (H_(3)_ = 25.43, *p* = 0.000). As shown in Figure 5a, a significant decrease (56.44%) in central crossing was observed in the 6‐OHDA‐lesioned group versus the control‐operated rats(*p* = 0.002). Administration of cedrol (10 mg/kg and 20 mg/kg) significantly increased central crossing (100% and 129.6%, respectively) (*p* = 0.008, and *p* = 0.001, respectively) as compared to the 6‐OHDA‐lesioned group. Central time also significantly diminished (64.15%) in the 6‐OHDA‐lesioned group versus the control‐operated animals(*p* = 0.000). Cedrol at 20 mg/kg dose significantly increased central time (85.80%) versus the Parkinson group (*p* = 0.026) (Figure 5b). Central distance significantly diminished (73.41%) in the PD group versus the control‐operated animals(*p* = 0.000). Administration of either dose of cedrol significantly increased central distance (142.39% and 177.56%) versus the PD group (*p* = 0.027 and *p* = 0.002) (Figure 5c).

**FIGURE 6:**
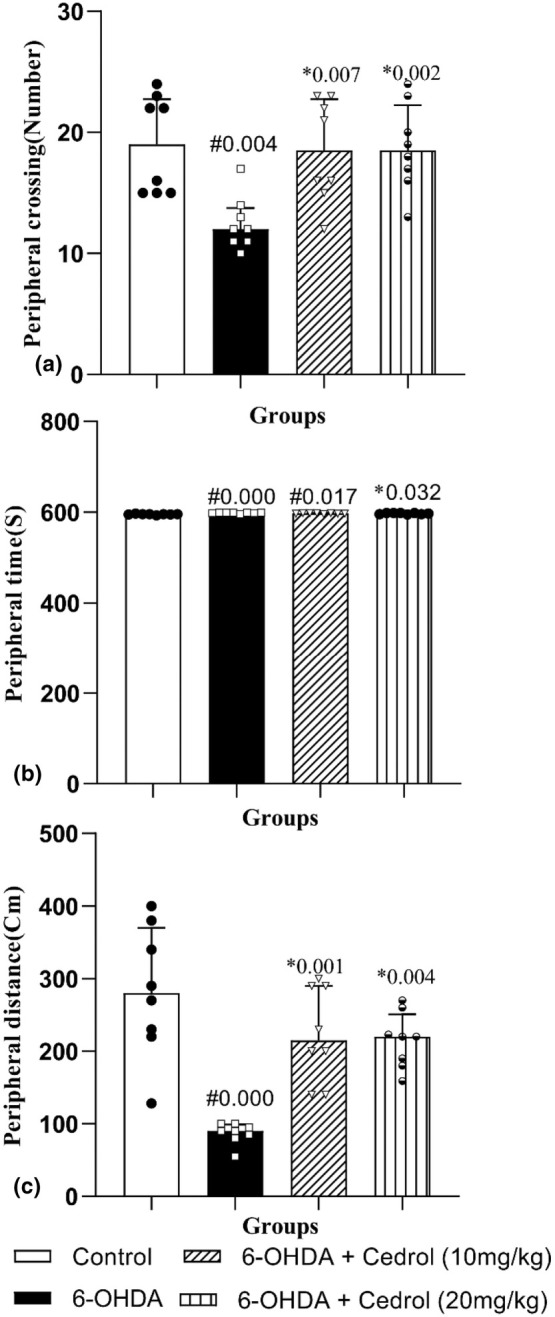
The effects of cedrol on the peripheral crossing number (a), the time spent in the peripheral zone (b), and the peripheral traveled distance (c) of the open field test. #Compared to the control group. *Compared to the Parkinson's group. The data were presented as median and interquartile range (*n* = 8 in each group).

The results showed that there was a significant difference between the groups on peripheral crossing (H_(3)_ = 12.62, *p* = 0.006), peripheral time (H_(3)_ = 16.61, *p* = 0.001), and peripheral distance (H_(3)_ = 19.43, *p* = 0.000). As shown in Figure 6a, a significant decrease in peripheral crossing (34. 21%) was observed in the 6‐OHDA‐lesioned group versus the control‐operated rats (*p* = 0.004). Administration of cedrol (10 mg/kg and 20 mg/kg) significantly increased peripheral crossing (48% and 50%) as compared to the 6‐OHDA‐lesioned group (*p* = 0.007, and *p* = 0.002, respectively) (Figure 6a).

Peripheral time significantly increased (0.48%) in the PD group versus the control‐operated animals (*p* = 0.000). Administration of 20 mg/kg cedrol significantly decreased peripheral time (0.22%) versus the Parkinson group (*p* = 0.032) (Figure 5b). Peripheral distance significantly diminished (69.22) in the PD group versus the control‐operated animals (*p* = 0.000). Administration of either dose of cedrol significantly increased central, peripheral, and total distance (157.56% and 147.78%) versus the PD group (*p* = 0.001 and *p* = 0.004) (Figure 6c).

**FIGURE 7:**
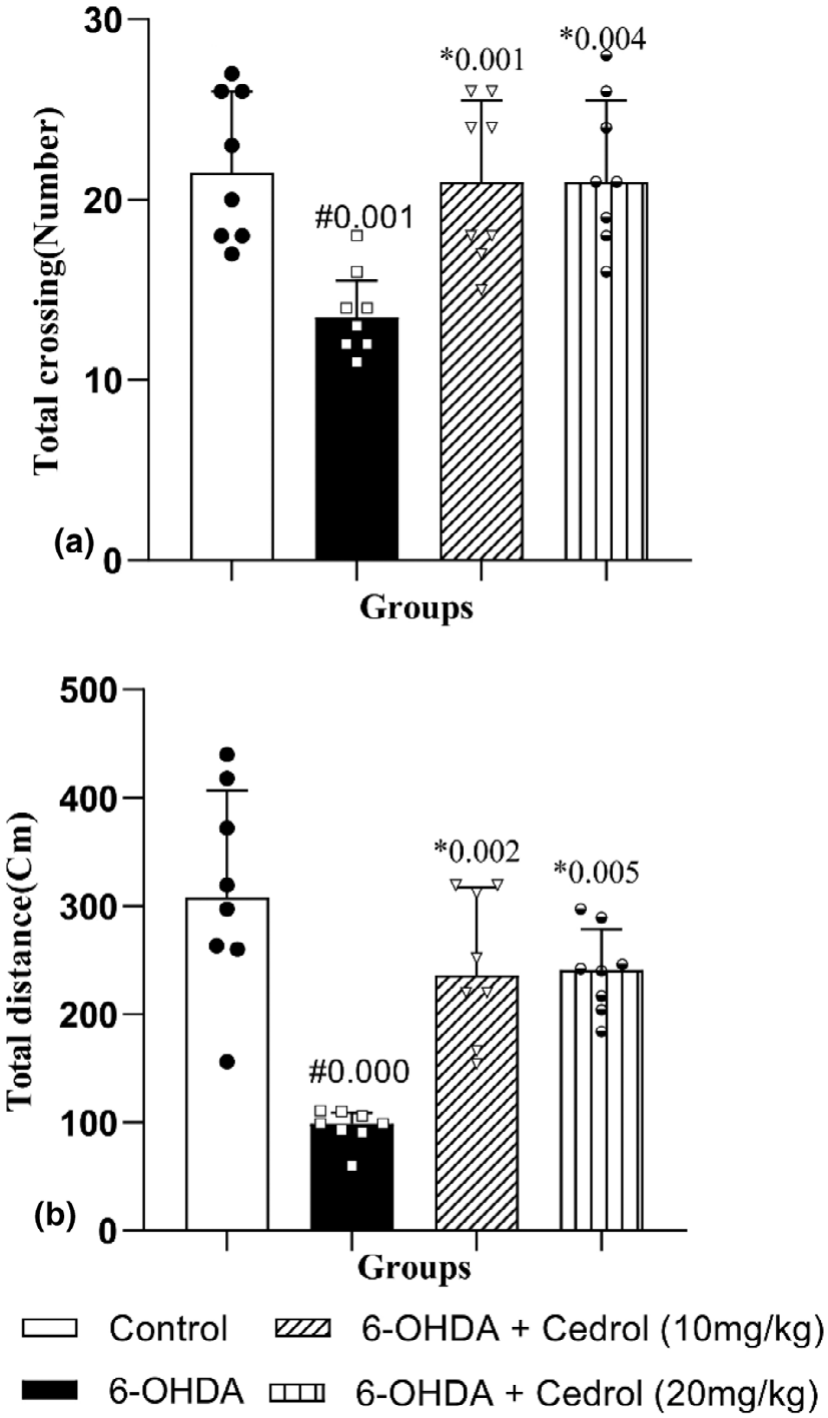
The effects of cedrol on the total crossing number (a) and total traveled distance (b) of the open field test. #Compared to the control group. *Compared to the Parkinson's group. The data were presented as median and interquartile range (*n* = 8 in each group).

The results showed that there was a significant difference among the groups on total crossing (H_(3)_ = 15.09, *p* = 0.002), and total distance (H_(3)_ = 20.12, *p* = 0.000). Total crossing significantly diminished (37.12%) in the PD group versus the control‐operated animals (*p* = 0.001). Administration of either dose of cedrol significantly increased total crossing (52.72% and 57.23%) versus the Parkinson group (*p* = 0.001 and *p* = 0.004) (Figure 7a). Total distance also significantly diminished (69.65%) in the PD group versus the control‐operated animals(*p* = 0.000). Administration of either dose of cedrol significantly increased total distance (155.14% and 149.55%) versus the PD group (*p* = 0.002 and *p* = 0.005) (Figure 7b).

### 3.5 Oxidative stress markers

The results showed that there was a significant difference between the groups on MDA (*F*_(3,20)_ =16.84, *p* = 0.000), total thiol (*F*_(3,20)_ =25.62, *p* = 0.000), and SOD (*F*_(3,20)_ =13.84, *p* = 0.000).

**FIGURE 8:**
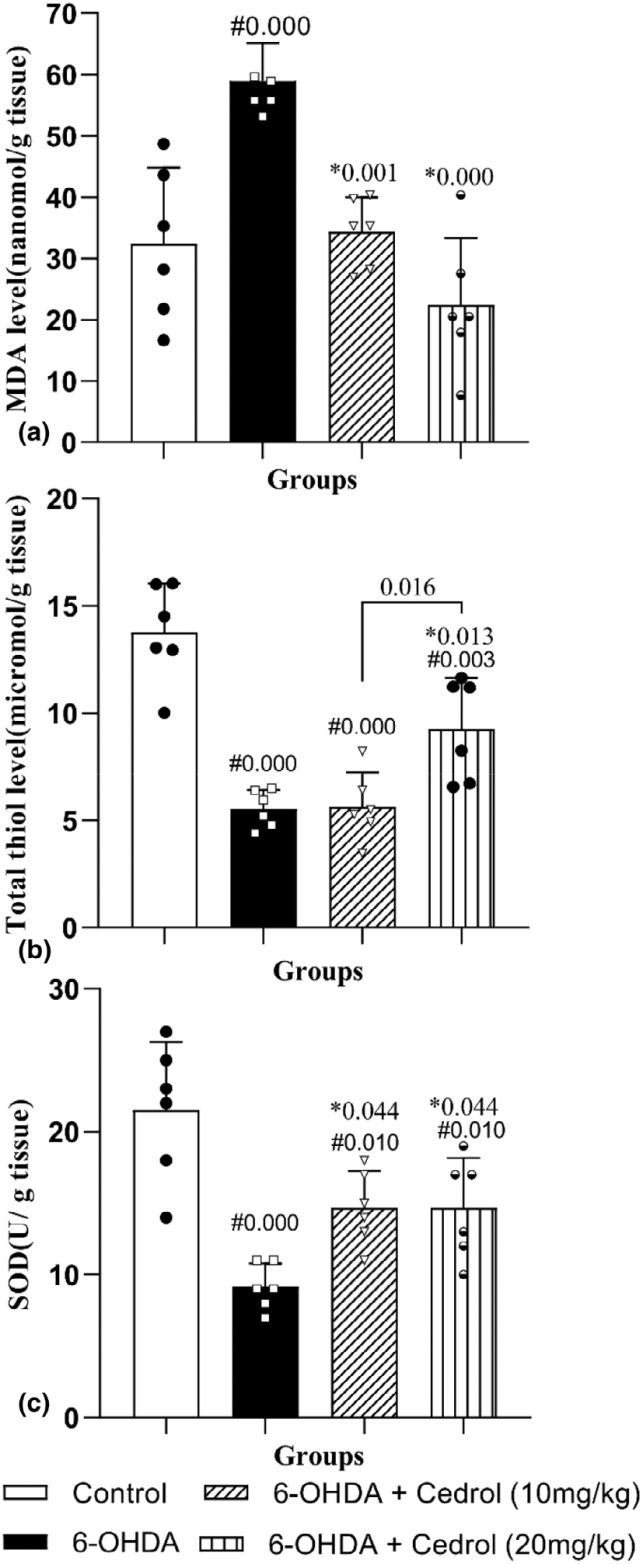
The effects of cedrol on MDA (a), total thiol levels (b), and SOD activity (c). #Compared to the control group. *Compared to the Parkinson's group. The data were presented as mean ± SD (*n* = 6 in each group). MDA, malondialdehyde; SOD, super oxidase dismutase.

Induction of the lesion significantly enhanced the level of MDA in the striatal tissue (82.17%) (PD group vs. control group, *p* = 0.001). The amounts of MDA were significantly lower in both treatment groups (*p* = 0.001 and 0.000) versus the PD group (41.83% and 61.96%) (Figure 8a). In addition, a significantly lower level of total thiol was found in the striatal tissues of the PD group rats versus the control‐operated rats (59.73%) (*p* = 0.000). Administration of the high dose of cedrol significantly reversed the attenuated level of total thiol (67.32%) (high‐dose‐treated group vs. PD group, *p* = 0.013) (Figure 8b). Induction of the lesion significantly (*p* = 0.001) reduced the activity of SOD in the striatal tissue when compared to control‐operated rats (57.39%), which was restored significantly in both treatment groups as compared to the lesion group (60.04%) (*p* = 0.044) (Figure 8c).

## 4 DISCUSSION

In this study, we found promising results for cedrol in the treatment of PD in a rat model, as measured by behavioral tests and oxidative stress biomarkers. The induction of PD adversely affected movement and cognitive function in the experiment animals. It also increases oxidative stress. Treatment with cedrol significantly ameliorated the alterations in motor and cognitive functions and reversed the altered levels of oxidative stress biomarkers.

Experimental animal models of neurodegenerative disorders investigate the mechanisms of disease and are used to develop new treatment strategies (Noor et al., 2016). Various animal models of PD have been introduced, including neurotoxin, genetic, and combined models (Kin et al., 2019). The 6‐OHDA lesion of the rat nigrostriatal pathway is the most commonly used animal model of PD (Torres & Dunnett, 2012). Due to its inability to penetrate the blood–brain barrier, 6‐OHDA must be directly injected into the brain tissue (Kin et al., 2019). The similarity of 6‐OHDA to catecholamines results in the neuronal uptake of this neurotoxin by dopamine and norepinephrine membrane transporters (Prasad & Hung, 2020).

In the present study, this model was induced by the unilateral injection of 6‐OHDA into the MFB. The subsequent generation of ROS, caused by the administration of this neurotoxin, damages dopaminergic neurons and induces PD. Injection of 6‐OHDA into this site resulted in the impairment of mitochondrial function in the substantia nigra pars compacta and the necrosis of dopaminergic neurons (More et al., 2016).

The dopamine receptor agonists, apomorphine, function postsynaptically and cause rotation to occur in the opposite contralateral direction—that is, away from the side that has been lesioned by hyperstimulating supersensitive dopamine receptors in the denervated striatum (Björklund & Dunnett, 2019).

In preclinical research, the “rotarod” technique has shown to be very beneficial for assessing medications that impact motor coordination. The rotarod test involves forced motor activity—typically by rodents—on a long, cylindrical spinning rod. This test is used to measure the rodents' motor coordination, balance, and grip strength, particularly when testing experimental medications in animals with movement problems (Jamwal et al., 2017).

The rotarod test measures the time the rats can maintain on a rotating rod. We utilized the apomorphine‐induced rotational test and the rotarod test to assess motor function and found that the administration of cedrol could significantly reduce the number of contralateral rotations and increase the duration spent on the rotating rods versus the lesioned group. Furthermore, we took advantage of the passive avoidance test, a commonly used method for evaluating cognitive function. In this test, normal healthy rats should avoid entering the chamber of a Shuttle box, where they received a shock upon their first entrance in the training trial (Bigham et al., 2021; Ögren & Stiedl, 2010). Treatment with cedrol significantly increased the delay before entering the dark space and the time spent in the lit chamber. Furthermore, the open field test was conducted to examine locomotor activity and behaviors related to anxiety (Su et al., 2018). Numerous parameters were assessed in the test, and obvious decreases in total crossing, center crossing, peripheral crossing, central time, and an increase in peripheral time were observed in the 6‐OHDA‐lesioned group versus the control group.

A substantial amount of evidence at the cellular level corroborated that “oxidative stress” leads to the loss of nigral dopamine‐producing neurons. Reactive oxygen and nitrogen species (ROS/RNS) build up intracellularly as a result of either an excess of ROS produced by the cell or a decrease in the body's natural antioxidant capacity. This condition is known as oxidative stress. Since mitochondria produce ROS, mainly superoxide and hydrogen peroxide, during respiration, it follows that all aerobic organisms are vulnerable to oxidative stress. The brain is vulnerable to oxidative damage because it uses 20% of the body's oxygen supply and is rich in fatty acids that are more readily peroxidized, while the antioxidant defenses of the brain, including catalase, glutathione peroxidase, glutathione, and SOD are quite spare (Niranjan, 2014).

Because the substantia nigra functions in a prooxidative state in comparison to other brain regions‐even in healthy individuals‐it is crucial that this region appears to be among the most vulnerable. Specifically, the substantia nigra has a low concentration of antioxidants (glutathione in particular), a high content of oxidizable species such as ROS, and a high metabolic rate, all of which make this area of the brain extremely susceptible to the effects of peroxynitrite and sulfite (Niranjan, 2014).

MDA is a hazardous aldehyde that is reactive and is produced when polyunsaturated fatty acid (PUFA) peroxide and certain lipid peroxide (LPO) products break down. MDA can combine with nucleic acid bases to generate stable protein adducts that further induce powerful immune responses and have pro‐inflammatory qualities. Moreover, MDA accumulation has the power to alter membrane permeability and reduce the fluidity of the membrane lipid bilayer (Taso et al., 2019).

The first line of defense against reactive oxygen species is typically thought to be SODs, due to their capacity to convert superoxide radicals to molecular oxygen and hydrogen peroxide (De Lazzari et al., 2018). The organic substances with a sulfhydryl group are called thiols. Thiols make up the majority of the body's total antioxidant supply and are essential for protecting the body from reactive oxygen species, among all other antioxidants. Total thiols are made up of intracellular and extracellular thiols that are either bound to proteins or exist in the free form as reduced or oxidized glutathione (Prakash et al., 2009).

In the current study, we measured MDA, SOD, and total thiol as markers of oxidative stress in the striatum. Treatment with cedrol significantly attenuated the levels of MDA and enhanced the levels of total thiol and SOD activity in the striatum, compared to the non‐treated lesioned rats.

Administration of cedrol in animal models of neuropathic pain and rheumatoid arthritis reduced oxidative stress and inflammation as marked by reduction of MDA, tumor necrosis factor (TNF)‐a, interleukin (IL)‐1β, and increment of total thiol level (Forouzanfar et al., 2022; Sakhaee et al., 2020). Despite the beneficial effects of cedrol in PD through its antioxidant features, in a study by Zhang and colleagues, the level of dopamine was found to be attenuated in female mice after the administration of cedrol (Zhang & Yao, 2019).

However, SCH23390, a dopamine D1 receptor antagonist, was used in a more recent study by this team, which demonstrated that the dopamine D1 receptor is implicated in the effects of cedrol in the brain, as administration of SCH23390, suppressed the anxiolytic impact of cedrol (Zhang et al., 2020).

In the nervous system of humans, D1 receptors are the most prevalent type of dopamine receptors. The stimulation of the dopamine D1 receptor activates the direct pathway in the striatum, a pathway whose activity is impaired in PD (Latif et al., 2021; Morelli et al., 2012). Hence, future research could assess the alterations of dopamine levels in specific brain regions involved in the course of PD. Further investigations could also be done to elucidate whether cedrol induces histological changes in the SNpc and striatum.

In previous studies, sesquiterpene compounds have been shown to have protective effects in PD.

In a study, reynosin, a sesquiterpene lactone, decreased 6‐OHDA‐induced cell death in SH‐SY5Y cells. Also, reynosin increased tyrosine hydroxylase‐positive cells in the in vivo model of PD. Furthermore, reynosin reduced the overexpression of α‐synuclein protein in in vivo and in vitro models of PD (Ham et al., 2013). Isolongifolene, a sesquiterpenes compound, reduced oxidative stress, motor impairment, and apoptosis in the rotenone‐induced PD model (Balakrishnan et al., 2021). Another sesquiterpene named β‐Caryophyllene reduced proinflammatory cytokines and oxidative stress markers (reduction of lipid peroxidation and increment of SOD and CAT activities) in the in vivo model of PD in rats (Ojha et al., 2016).

Our manuscript has some limitations: First, we started administering cedrol 3 days before inducing the model; second, we did not measure dopamine levels.

## 5 CONCLUSION

In this study, cedrol alleviated movement and cognitive impairments in the rat in vivo model of PD. We can assume that the protective benefits of cedrol may be linked to the reduction of oxidative stress because our study's results demonstrated an improvement in the levels of oxidative markers in PD rats treated with cedrol.

## AUTHOR CONTRIBUTIONS

F. F., M. H., and A. M. P‐Sh. performed the experiments. F. F., M. H., and A. M. A. participated in writing the paper. F. F. was the project's supervisor and prepared the paper's final draft.

## FUNDING INFORMATION

This manuscript was supported by grants from MUMS, Mashhad, Iran (No: 4010185, IR.MUMS.AEC.1401.039).

## CONFLICT OF INTEREST STATEMENT

All authors manifest no conflict of interest.

## ETHICS STATEMENT

The use of the rats was designed and performed in compliance with the National Institutes of Health guidelines for the Care and Use of Laboratory Animals. The study was approved by the Ethical Committee of MUMS (No: 4010185, IR.MUMS.AEC.1401.039).

## Data Availability

The datasets of the current study are available from the corresponding author on request.
